# Acute Pancreatitis Induced Splenic Vein Thrombosis

**DOI:** 10.7759/cureus.15714

**Published:** 2021-06-17

**Authors:** Chidinma Ejikeme, Sherif Elkattawy, Fisayo Kayode-Ajala, Abraheim Al-nasseri, Arun Naik

**Affiliations:** 1 Internal Medicine, Rutgers New Jersey Medical School/Trinitas Regional Medical Center, Elizabeth, USA; 2 Internal Medicine, Trinitas Regional Medical Center, Elizabeth, USA; 3 Internal Medicine, St. George's University School of Medicine, Elizabeth, USA; 4 Gastroenterology, Trinitas Regional Medical Center, Elizabeth, USA

**Keywords:** pancreatitis, splenic vein thrombosis, complications, pancreatic exocrine insufficiency, inflammation

## Abstract

Acute inflammation of the pancreas, known as pancreatitis, can result in many complications ranging from acute distress respiratory syndrome to pancreatic necrosis. A relatively common vascular complication of pancreatitis is splenic vein thrombosis (SVT) due to intimal inflammation leading to platelet aggregation and thrombosis. The management of SVT with regard to anticoagulation (AC) might appear to be perplexing at first given the recommendation to withhold any sort of AC. Research studies have shown that these patients have an increased risk of gastrointestinal bleeding without AC. In this report, we discuss a case of hypertriglyceridemia-induced pancreatitis. During hospitalization, our patient complained of worsening abdominal pain with objective fevers and leukocytosis in which CT scan of the abdomen was significant for hemorrhagic pancreatitis with necrosis, acute SVT, and splenomegaly. The patient was managed conservatively with IV fluids, pain relief medications, and antibiotics.

## Introduction

Pancreatitis by definition refers to an acute or chronic inflammation of the pancreas. Chronic pancreatitis can result in permanent structural changes which can lead to exocrine and endocrine dysfunction [1]. Pancreatitis complications include pancreatic duct dysfunction, obstruction, and vascular complications [2-3]. We present a case of a 28-year-old male with a past medical history (PMH) of type I diabetes mellitus, and alcohol abuse who developed acute splenic vein thrombosis (SVT) secondary to interstitial edematous pancreatitis. SVT represents one of the vascular complications of chronic pancreatitis [2]. Patients with this complication typically are asymptomatic, however, symptomatic patients usually present with gastric varices and evidence of splenomegaly on physical examination [3]. Our case will highlight the development of SVT post-pancreatitis inflammation.

## Case presentation

**Figure 1:**
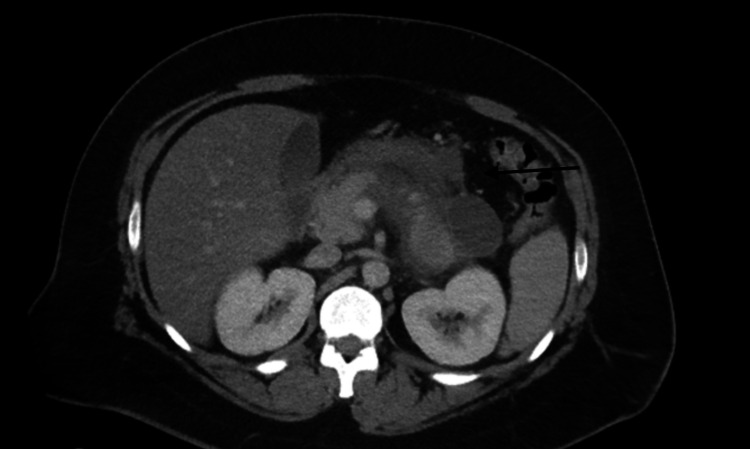
CT scan of the abdomen without contrast revealed acute interstitial edematous pancreatitis primarily involving the pancreas body and tail.

A 28-year-old male with a past medical history (PMH) of type I diabetes mellitus and alcohol abuse presented to the emergency department (ED) with complaints of abdominal pain. His symptoms started on awakening that morning. He described it as a sudden onset epigastric pain, rated 10/10 and non-radiating. The patient also became nauseous and had episodes of non-bilious, non-bloody vomiting, which prompted his ED visit. The patient denied headaches, dizziness, chest pain, shortness of breath, diarrhea, dysuria, or neurological changes. In the ED, vital signs were remarkable for a temperature of 98.9°F, heart rate of 110 bpm, respiratory rate of 15 breaths/min, and blood pressure (bp) of 160/111 mmHg. Labs were remarkable for white cell count 20.3 k/uL, hemoglobin 17.4 g/dL, and serum lipase level 665 u/L. The lipid panel revealed a total cholesterol level of 1154 mg/dL, high density lipoprotein (HDL) of 23 mg/dl, with significantly elevated low density lipoprotein (LDL) and triglyceride level, such that it was too high to calculate due to lipemia. A CT scan of the abdomen without contrast revealed acute interstitial edematous pancreatitis primarily involving the pancreas body and tail. There was also acute peripancreatic fluid collection measuring 7 cm x 4 cm x 5 cm as seen in Figure 1.

Of note, the patient was admitted in 2019 with similar symptoms where he was diagnosed with hypertriglyceridemia-induced pancreatitis and was discharged on atorvastatin and gemfibrozil. During this evaluation, the patient stated that he is non-compliant with medications and that he now drinks an average of four cans of beer twice weekly.

**Figure 2:**
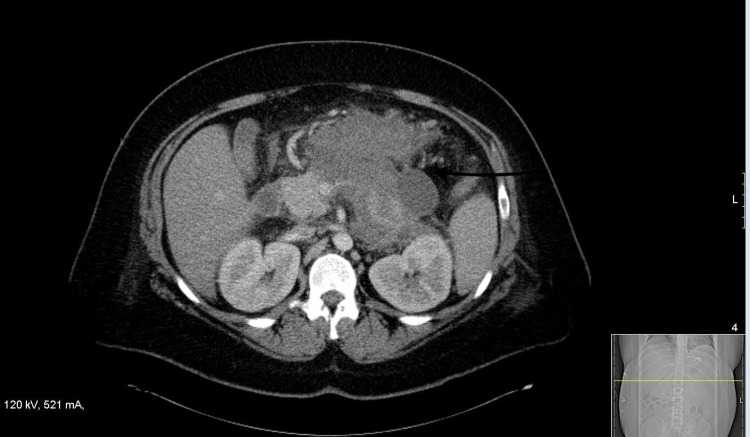
CT scan of the abdomen significant for extensive hyperdense peripancreatic fluid, consistent with hemorrhagic pancreatitis.

**Figure 3:**
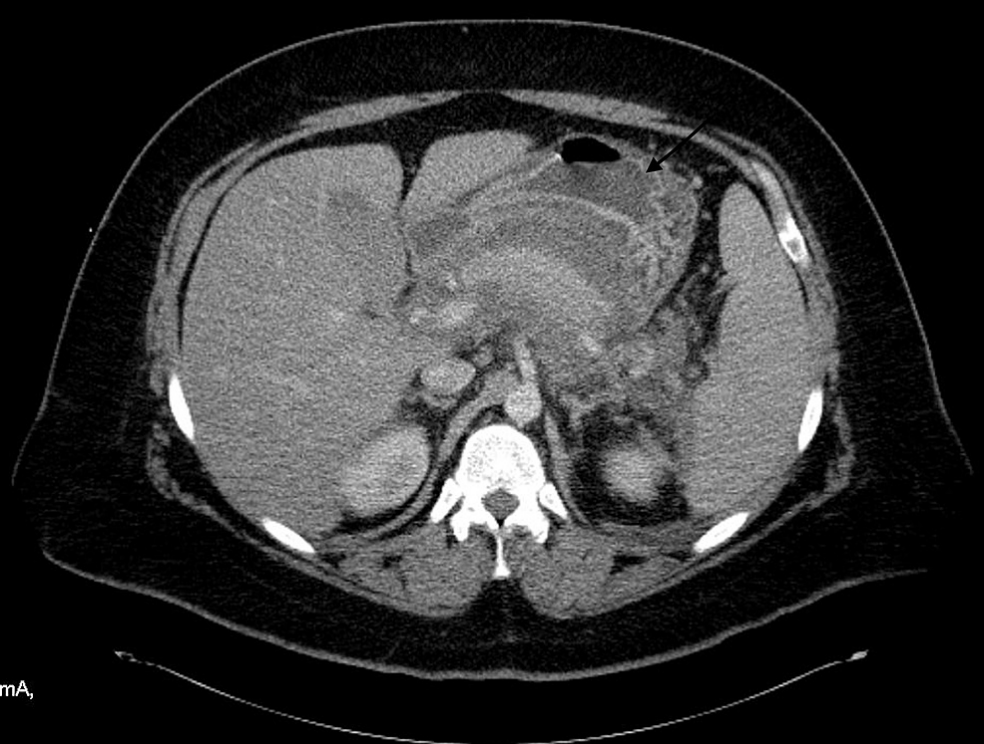
CT scan of the abdomen shows new areas of non-enhancement within the pancreatic tail, compatible with necrosis.

Given hypertriglyceridemia, the patient was placed on strict nil per os (NPO) and transferred to the ICU for further management. IV fluid and insulin infusion were initiated for the management of recurrent hypertriglyceridemia pancreatitis. Frequent blood work was performed to monitor lipid panel and potassium level while on insulin infusion. About three days following treatment, the patient's symptoms started to improve on the current treatment. Repeat lipid panel showed an improvement of triglyceride level at 470 mg/dL, LDL of 177 mg/dL, and total cholesterol of 284 mg/dL. He reported having minimal to no abdominal pain; hence he was advanced to a clear liquid diet. He was placed on a low-fat diet, atorvastatin, and gemfibrozil for better lipemic control. While on current management, the patient's abdominal pain started to worsen again. He began to have multiple episodes of fever. His white cell count started to increase significantly, and his hemoglobin level decreased to 12.7 g/dL. A repeat CT abdomen was performed, which revealed an extensive hyperdense peripancreatic fluid with findings suggestive of hemorrhagic pancreatitis, with acute SVT and splenomegaly as seen in Figure 2. There were also new areas of non-enhancement within the pancreatic tail compatible with necrosis as seen in Figure 3.

The patient was started on meropenem, and hemoglobin levels were monitored frequently. The hepatobiliary surgery team was consulted, who recommended supportive management with antibiotics, IV fluid, and to advance diet as tolerated. Blood cultures were performed, which were negative. The patient’s symptoms eventually improved and his hemoglobin levels stabilized. He was gradually restarted on a consistent carbohydrate diet without any new complications. The patient was discharged with lipid-lowering medications and scheduled for an outpatient appointment. 

## Discussion

The management of our patient’s pancreatitis-induced splenic vein thrombosis (PIVST) was mostly supportive measures with significant improvement of symptoms. Inflammation of the pancreas may lead to inflammation of the underlying splenic vein, leading to thrombus formation and other pathological sequelae (i.e., left-sided portal hypertension and gastric varices) [4-5]. Since acute inflammation encompasses the capacity to resolve, treatment of the underlying pancreatic inflammation eventually eliminates complications associated with splenic vein inflammation [6-9]. An understanding of the pathophysiology involved in PISVT supports our approach in management and simplifies the treatment strategy for select patients (young patients with gradual improvement of acute pancreatitis (AP) under standard therapy, and have no history of bleeding diatheses). 

In addition to the pathophysiology, clinical research also supports standard AP treatment as a means of managing PIVST in select patients [5]. Gonzelez et al. reported that no significant differences were observed in AP-induced splanchnic vein recanalization, irrespective of whether or not they received anticoagulation (AC) therapy (p = 0.076). Furthermore, the study concluded that the self-limited treatment for AP with splanchnic thrombosis could be a result of the self-limiting resolution of AP and/or drainage of adjacent collections [1]. AC may also be a relative contradiction when treating PIVST, as a cohort study by Anderson et al. concluded that AC significantly increased the risk for gastrointestinal (GI) bleeding (p = 0.032).2 PIVST already holds a 12.3% increased risk of GI bleeding without AC, this becomes especially useful for practicing evidence-based medicine (i.e., assessing the risk of hemorrhage during pancreatic surgery in a patient diagnosed with PIVST) [3,6]. In support of utilizing CT imaging to monitor our patient’s disease course, many researchers have proven the CT scan to be statistically significant in the accurate prediction of pancreatitis severity and prevalence of SVT [4, 7-8]. This becomes especially useful when determining whether or not self-limited management of PISVT is clinically useful in selective patients. 

As seen in our case, PISVT is a clinically relevant complication of AP and it is imperative to delineate the management strategy for these patients. Although several complications may arise in AP, it is imperative to differentiate between patients that only require a standard approach to care, from those that require advanced medical management [i.e., acute respiratory distress syndrome (ARDS) secondary to AP]. However, the treatment of PISVT must not be oversimplified, as standard management entails critical monitoring of the disease course during hospitalization. Additionally, some reports have determined splenomegaly as an asymptomatic sign of PIVST that may require splenectomy prior to pancreatic surgery [3, 10]. Further studies are needed to support the use of self-limited treatment for PISVT while stratifying patient demographics (including age, race, frequency of AP, and comorbidities). In addition, studies that measure the severity of portal vein thrombosis associated with PISVT, in comparison to PISVT alone, may be clinically significant in determining prognosis and providing more insight into the risks and benefits of treatment.

## Conclusions

The incidence of PISVT is estimated to be about 12% in the general population. In our case, our patient’s symptoms were self-limiting and resolved with standard treatment for pancreatitis. Further research is still needed to ascertain the benefits of self-limited treatment in addition to AC in PISVT patients.
